# Evaluation of bronchial hyperresponsiveness in asthmatic paediatric patients using mannitol challenge test – Impacts of body mass index

**DOI:** 10.1080/07853890.2025.2468262

**Published:** 2025-02-20

**Authors:** Sharon S. Y. Leung, Helen S. L. Tsang, Jasmine Chan, Oliver Y. H. Kui, Ping Zeng, Yin Ting Cheung, James-Wesley Cheng, Kate C. C. Chan, Michelle Yu, Patricia Tang, John D. Brannan, Jenny K. W. Lam, Hak-Kim Chan, Albert M. Li

**Affiliations:** a School of Pharmacy, The Chinese University of Hong Kong, Hong Kong SAR, China; b Department of Paediatrics, Prince of Wales Hospital, The Chinese University of Hong Kong, Hong Kong SAR, China; c Sydney Pharmacy School, University of Sydney, Sydney, Australia; d Department of Respiratory and Sleep Medicine, John Hunter Hospital, Newcastle, Australia; e Department of Pharmaceutics, UCL School of Pharmacy, University College London, London, UK

**Keywords:** Paediatric asthma, asthma control, mannitol challenge test, bronchial hyperresponsiveness, body mass index

## Abstract

**Background:**

Increasing epidemiological studies reported that overweight/obese asthma patients had more frequent and severe symptoms and exacerbations, indicating their asthma management may not be sufficient. Airway hyperresponsiveness (AHR), a significant feature of asthma, was found to link with the body mass index (BMI) with mixed findings using the “direct” methacholine challenge test. The objective was to examine the association between BMI and asthma control, as reflected by the “indirect” AHR with the mannitol challenge test in a paediatric asthmatic population.

**Methods:**

A total of 80 subjects with physician-diagnosed asthma, aged 6–18 years were enrolled in this cross-sectional study. Patients were first asked to complete the Asthma Control Test (ACT) questionnaire to self-evaluate their disease status. A mannitol challenge test was then performed to assess their AHR severity.

**Results:**

Seventy-six patients (96%) rated their asthma as well-controlled with an ACT score ≥ 20, but 42 patients (53%) were tested positive in the mannitol challenge test with mild and moderate AHR. While patients with mild AHR had comparable lung functions to those without AHR, patients with moderate AHR showed slightly but significantly lower FEV_1_ and FEV_1_/FVC values. Although no significant difference in the BMI values was noted for patients with different levels of AHR, the trend of increasing BMI with age was steeper for patients with moderate AHR.

**Conclusion:**

A high prevalence of AHR (>50%) was found in asthmatic children who self-evaluated with good asthma control. No significant influence of the BMI on the AHR severity could be demonstrated in this population with the “indirect” mannitol challenge test. Since only a small number of overweight/obese subjects were recruited in the present study, further verification of the results with a larger sample size of obese subjects is required.

## Introduction

The prevalence of childhood asthma and overweight/obesity has significantly increased in the past decades. Asthma is a common chronic lower respiratory disease in childhood characterised by inflammation, bronchoconstriction and airway hyperresponsiveness (AHR). It occurs in children and adults, affecting approximately 300 million individuals worldwide [1]. Being overweight/obese, defined as abnormal or excessive fat accumulation that may impair health, is another worldwide chronic health issue. According to the World Health Organization [2], about 340 million children and adolescents aged 5–19 years old are overweight/obese, and 39 million overweight/obese children are under five, with almost half of them living in Asia. Emerging evidence is supporting the positive association between increasing body mass index (BMI) and subsequent asthma development in children and adolescents [3,4]. In addition, previous epidemiological studies reported that overweight/obese asthma patients had more frequent hospitalisations for acute asthma and unscheduled doctor visits and lower quality of life compared with asthmatics with normal weight [5,6]. This indicates that the current asthma management strategies for overweight/obese asthmatic patients might not be sufficient.

According to the Global Initiative for Asthma [7], asthma control is assessed according to the frequency and intensity of daytime and nocturnal symptoms, activity limitation and frequency of reliever medication use. Recent studies showed that 45%–52% of patients with asthma in developed countries remains largely uncontrolled [8]. The asthma Control Test (ACT), a 5-item patient-administered survey for assessing asthma control, is often used as routine care by asthma specialists [9]. However, the ACT relies on patients’ self-perception of symptoms, which may vary considerably among patients and not reflect the actual clinical status [10,11]. Therefore, pulmonary function test, mostly spirometry, is sometimes used as an objective measurement of asthma control in clinical settings. However, it does not include an assessment of airway inflammation, which is asthma’s main primary pathogenic mechanism. In addition, previous studies have shown that asthmatic patients with underlying airway inflammation might still be at risk of severe exacerbations despite their symptoms being adequately controlled [12,13]. Therefore, recent GINA guidelines [7] also emphasise the importance of having laboratory biomarkers of airway inflammation and pathophysiological features of this disease in assessing asthma control. In this context, measuring AHR, which correlates with markers of airway inflammation and a sensitive airway smooth muscle, is more relevant in asthma control assessment [14,15].

AHR can be assessed using "direct" stimuli (such as histamine and methacholine) that act directly through receptors on the airway smooth muscles to induce bronchoconstriction or "indirect" stimuli (such as exercise, hypertonic saline and mannitol) that provoke bronchoconstriction *via* activating inflammatory cells to release inflammatory mediators [15]. Therefore, AHR in response to an indirect stimulus is believed to produce a more positive correlation with the underlying airway inflammation than the direct AHR [16]. Conventionally, inhaled bronchial provocation tests (BPT) are used for diagnostic investigation of patients with symptoms suggestive of asthma but show normal pulmonary function and no bronchodilator reversibility. BPT performed with mannitol have recently been suggested as a potential tests in evaluating the effect of anti-inflammatory treatments and the level of asthma control [17,18].

There have been increasing epidemiological studies showing a positive association between the development of asthma and obesity in patients of different age ranges [19]. In the paediatric population, a meta-analysis of case-control studies highlighted that being overweight or obese increased the risk of asthma by 1.64 and 1.92, respectively [20,21]. Obese asthmatic patients also tend to have increased asthma severity with decreased response to asthma medication and poorer asthma control, having more symptoms, and more frequent and severe exacerbations. Obese children hospitalised for asthma were also reported to be associated with longer length of stay and a higher risk of mechanical ventilation [22]. Obesity has, therefore, been considered a risk factor and a disease modifier of asthma in both children and adults [23]. However, the underlying mechanisms of their association remain unclear. Several hypotheses have been proposed, including obesity-induced systemic inflammation extending to airways, metabolic dysregulation, hormonal abnormalities and the mechanical effects of excess adipose tissue on the chest wall and abdomen causing compressed airways and contributing to AHR [20,21].

The relationship between BMI and AHR based on the “direct” methacholine challenge test was evaluated in various cohort studies with mixed findings [24–28]. Ciprandi et al. [27] evaluated the impact of BMI on the AHR of asthma patients in a cohort of Army Navy subjects. They found that the BMI was significantly higher for patients with severe AHR. Similar findings were reported in another cohort study with patients aged 12–84 years old [26]. Differently, Mansell et al. [25] reported a relationship between overweight and baseline flow limitation, but no association between overweight and AHR was noted in asthmatic adolescents. Kwon et al. [28] even reported a negative correlation between BMI and AHR. To uncover the relationship between BMI and AHR in the paediatric asthmatic population, the present study employed the “indirect” mannitol challenge test to measure AHR in 6–18 years children with active asthma. A positive response to the mannitol challenge test would depend on the presence of inflammatory cells, the concentration of mediators and muscle responsiveness. It is a closer reflection of active airway inflammation and resembles most daily life stimuli provoking an asthma attack.

## Methods

### Study design

This was a cross-sectional study, and subjects aged 6–18 years diagnosed with asthma and under regular monitoring at the Prince of Wales Hospital were consecutively enrolled from December 2020 to December 2022 until the previously calculated needed sample of 80 subjects was reached. After evaluating their eligibility according to the inclusion criteria, patients were enrolled at their normal appointment time. Written consent was obtained from all parents, and verbal assent was obtained from all children. All eligible patients were interviewed regarding their use of anti-asthmatic medications, such as no treatment, inhaled corticosteroids (ICS), long-acting β2-agonists (LABA), short-acting β2-agonists (SABA) and leukotriene receptor antagonists (LTRA). Patients’ medication information was also extracted from the electronic health medical records.

According to the manufacturing guidelines, eligible patients were asked to withhold their medications before the test. Subjects attending the testing day were asked to complete the ACT (cACT was used for patients aged 6–11) and questioned to verify medication withholding before the test. Subjects were first evaluated by a respiratory physician on their asthma control, followed by spirometry and bronchial provocation tests (BPT) with the mannitol challenge test kit (Aridol^®^) from Pharmaxis Ltd. (NSW, Australia).

This study was approved by the Joint Chinese University of Hong Kong-New Territories East Cluster Clinical Research Ethics Committee (CREC Ref. No.: 2019.287) and conducted in compliance with the Declaration of Helsinki and ICH-GCP Guidance (ICH E6).

### Subjects and sampling size determination

Eighty subjects aged 6–18 years with physician-diagnosed asthma and classified as having active asthma based on the GINA guidelines were included in the study. Patients were excluded from participation if they (1) have a forced expiratory volume in 1 s (FEV_1_) less than 70% of the predicted value; (2) have other respiratory or obstructive diseases; (3) have an asthma exacerbation within the previous 4 weeks; or (4) have a respiratory tract infection within the previous 4 weeks. Few studies in the literature have investigated the impact of weight status on AHR in the pediatric population. Hence, the sample size calculation was based on a previous trial on a navy population reported an odd ratio of 4.5 between overweight/obesity and AHR positivity based on the methacholine challenge test [27]. Assuming the prevalence of the overweight/obese population in the local pediatric is 20% [29], a type 1 error (α) of 0.05 and a power (1-β) of 80%, the estimated sample size was 68 patients, according to Suresh and Chandrashekara [30].

### Body mass index (BMI)

BMI was calculated as the weight (kg) divided by the square of the height (m^2^) measured at the clinic. Patients were classified as underweight, normal weight, overweight and obese based on the BMI-for-age z-score according to a local reference 31: underweight (< −1.6449, corresponding to the <5^th^ percentile), normal (−1.6449 to 1.0364, corresponding to the 5th to 85th percentile), overweight (1.0364 to 1.6449, corresponding to 85th to 95th percentile) and obesity (>1.6449, corresponding to > 95th percentile).

### Spirometry

Spirometry was performed with a computer-assisted spirometer (GANSHORN SpiroScout, Deutschland, Germany) and according to international guidelines [32]. Briefly, three blows (every 5 mins) were performed, and the best result was considered. All subjects met the criteria for reproducibility and acceptability. All 80 subjects performed spirometry and confirmed FEV_1_ > 70% before the mannitol challenge test.

### Mannitol challenge test using aridol^®^

The mannitol inhalation powder (Aridol^®^), which is a quick (total test time < 1 h) and easy-to-use test kit, was used [33]. The test was performed in a doubling dose series: 0, 5, 10, 20, 40, 80, 160, 160, and 160 mg, with 635 mg as the maximum cumulative dose. The subjects were trained on the use of Aridol^®^ using an inspiratory flow meter configured to ensure the inspiratory flow rate ranged 30 – 50 L/min. Subjects were asked to repeat practicing to familiar themselves with the required inhalation effort. Spirometry was performed 1 min after inhalation of the 0 mg capsule to take the baseline FEV_1_. The test was terminated when (1) the FEV_1_ value fell ≥ 15% from the baseline value, (2) it fell by at least 10% between two consecutive doses, or (3) the highest concentration of mannitol had been given. A positive response to the mannitol challenge test was determined with the threshold dose causing a 15% fall of FEV_1_ (PD_15_). On completion of the challenge, all subjects received a dose of 200 μg salbutamol *via* a Compact Space Chamber Plus (Medical Developments International Ltd, Victoria, Australia). If the patients’ FEV_1_ value did not returned to within 5% of the baseline FEV_1_ in 15 min, a second dose of salbutamol was given.

### Degree of AHR

The provocative dose (PD_15_) was determined for all subjects. Four categories of AHR were classified according to Anderson [33] based on the PD_15 _dose: severe AHR ≤ 35 mg; moderate AHR 36–155 mg; mild AHR 156–635 and without AHR > 635 mg. The degree of airway reactivity to mannitol (percent fall in FEV_1_ divided by the final cumulative dose of mannitol), known as the response dose ratio (RDR), was also assessed and presented as RDR_100_ = RDR × 100 defined according to a previous study [34].

### Statistical analysis and data definitions

The only subject tested with a PD_15_ < 35 mg was grouped with the moderate AHR group for analysis. Descriptive statistics were first performed, and quantitative parameters were reported as means ± one standard deviation (SD). PD_15_ and RDR_100_ were analysed after log transformation and their geometric mean (Gmean) and 95% CI was detemined. Qualitative data were reported as absolute frequencies/percentages. The chi-square test was used to compare qualitative variables among groups with post-hoc comparison using Bonferroni’s correction. Comparison of quantitative variables among groups was made using the parametric analysis of variance (ANOVA) followed by Turkey’s multiple comparisons tests. This study analysed data with SPSS software (version 20). A *p* value less than 0.05 was considered statistically significant.

## Results

Demographic characteristics of recruited subjects, including gender, age, BMI, inhaled corticosteroid (ICS) usage, concomitantly with allergic rhinitis, baseline FEV_1_ and forced vital capacity (FVC) and their ratio, FEV_1_/FVC, are reported in Table 1. Eighty previously diagnosed asthmatic patients, 55 boys and 25 girls, with an average age of 11.1 ± 3.2 years, ranging from 6 to 18 years, were included in this study. The average BMI of the patients was 19.0 ± 4.3 kg/m^2^, with the computed BMI z-score spanning from −4.05 to 2.21. Detailed demographic characteristics of recruited patients are summarised in Table 1.

**Table 1.** Baseline characteristics of study subjects and their responses to the mannitol challenge test.

Variable	Total (*n* = 80)	Mannitol Challenge test		*p*
		Negative	Positive	
**Gender**				
Male	55 (68.8%)	26 (47.3%)	29 (52.7%)	.952^a^
Female	25 (31.3%)	12 (48.0%)	13 (52.0%)	
**Age (in years)**	11.1 (3.1)	11.4 (3.4)	10.8 (2.9)	
6–11 yr	49 (61.3%)	23 (46.9%)	26 (53.1%)	.899^a^
12–18 yr	31 (38.8%)	15 (48.4%)	16 (51.6%)	
**ICS use**				
Yes	24 (30%)	7 (29.2%)	17 (70.8%)	.032^a^
No	56 (70%)	31 (55.4%)	25 (44.6%)	
**With allergic rhinitis**				
Yes	68 (85%)	28 (41.2%)	40 (58.8%)	.007^a^
no	12 (15%)	10 (83.3%)	2 (16.7%)	
**Parental smoking**				
Yes	25 (31.3%)	9 (36.0%)	16 (64.0%)	.165^a^
no	55 (68.8%)	29 (52.7%)	26 (47.3%)	
**Spirometry**				
FEV_1_ %	98.0 (12.8)	100.6 (13.4)	95.6 (11.9)	.080^b^
FVC %	96.0 (11.6)	96.7 (12.4)	95.3 (10.8)	.590^b^
FEV_1_ /FVC	0.86 (0.07)	0.87 (0.06)	0.85 (0.09)	.150^b^
**BMI**	19.0 (4.3)	19.3 (4.2)	18.7 (4.5)	.150^b^
Underweight	10 (12.5%)	4 (40.0%)	6 (60.0%)	.763^a^
Normal	47 (58.8%)	21 (44.7%)	26 (55.3%)	
Overweight	11 (13.8%)	6 (54.5%)	5 (45.5%)	
Obese	12 (15.0%)	7 (58.3%)	5(41.7%)	

All variables are presented as mean (SD), except for gender, ICS user and allergic rhinitis, which is presented as number of patients *n* (%).

FEV_1_: forced expiratory volume in 1s; FVC: forced vital capacity.

^a^Chi-square test.

^b^ANOVA.

### Comparisons between ACT scores and mannitol challenge test outcomes

**Figure 1.**
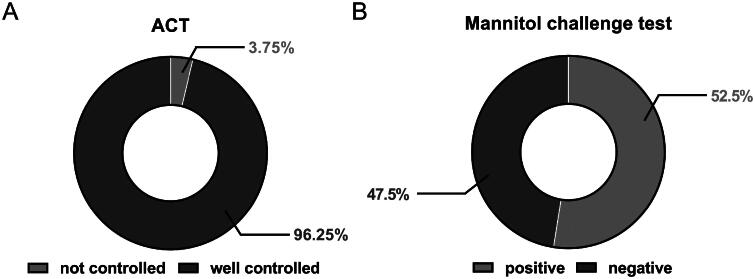
(A) ACT/cACT and (B) mannitol challenge results of recruited patients.

Prior to the mannitol test, patients were asked to self-evaluate their asthma control status by filling out the ACT/cACT. Seventy-seven patients (96%) rated themselves with well-controlled asthma with an ACT/cACT score ≥ 20. However, more than half of the recruited patients (53%) later tested positive for the mannitol challenge test (Figure 1). Out of the three patients who rated their asthma as uncontrolled, two tested positive for the mannitol challenge test.

### Effects of gender, age and medical conditions

The positive response rates to the mannitol challenge test for male (52.7%) and female (57.0%) patients were comparable (Table 1), suggesting there is no gender effect in the asthma control in this cohort. Due to the different capabilities in self-reporting their symptoms and using their inhaler medication for patients of varying age, we further examined patients’ mannitol challenge test outcomes in two groups according to their age, 6–11 yr and 12–18 yr, consistent with the age of patients using the cACT and ACT questionnaires. Table 1 shows no significant difference in the positive responses to the mannitol test (53.1% vs 51.6%).

As shown in Table 1, patients receiving anti-inflammatory treatment with ICS had a significantly higher rate of positive mannitol challenge test results compared with those who did not need ICS to control their asthma (70% vs. 45%). Also, a higher positive response rate was noted for patients with concomitant allergic rhinitis than those without (59% vs 17%).

### Baseline lung function and AHR severity

**Figure 2.**
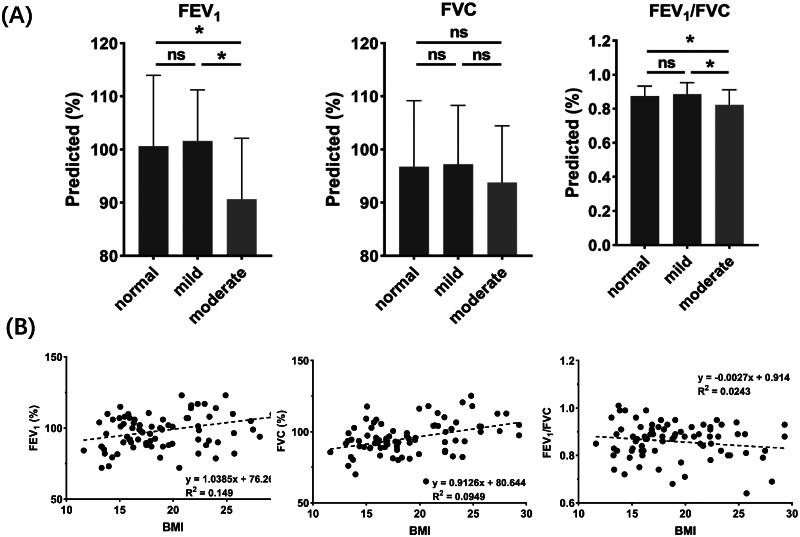
The baseline lung function of recruited patients as a function of (A) AHR severity and (B) BMI. Significant differences are indicated by asterisks (*p ≤ .05).

Table 1 shows no significant difference in the baseline spirometry characteristics (FEV_1_, FVC and FEV_1_/FVC) between patients with negative and positive responses to the mannitol challenge test. Figure 2A analyses the baseline spirometry characteristics of recruited patients based on their AHR severity. While the FEV_1_ values for patients with normal (Mean: 100.6; SD: 13.4) and mild (Mean: 101.6; SD: 9.6) AHR levels were comparable, they were significantly higher than patients with moderate AHR level (Mean: 90.6; SD: 12.7; *p* = .07 with respect to normal group and *p* = 0.012 with regard to mild group). The FVC values were comparable (Mean: 93.7–97.2; SD:10.7–12.4; *p* > .05) for patients with different degrees of AHR. Patients with moderate AHR levels were also associated with a lower overall FEV_1_/FVC ratio (Mean: 0.82; SD: 0.09) than those with normal (Mean: 0.87; SD: 0.06; *p* = .021) or mild (Mean: 0.88; SD: 0.07; *p* = .019) AHR levels. No significant FEV_1_/FVC ratio difference was found between patients with normal and mild AHR levels. These were consistent with previous reports [35,36].

Figure 2B shows the variation in lung function with BMI. The FEV_1_ and FVC show a slightly increasing trend with increasing BMI. On the other hand, the FEV_1_/FVC ratio gradually decreased with increasing BMI. A similar observation was reported previously [28,36].

### BMI on AHR severity

**Figure 3.**
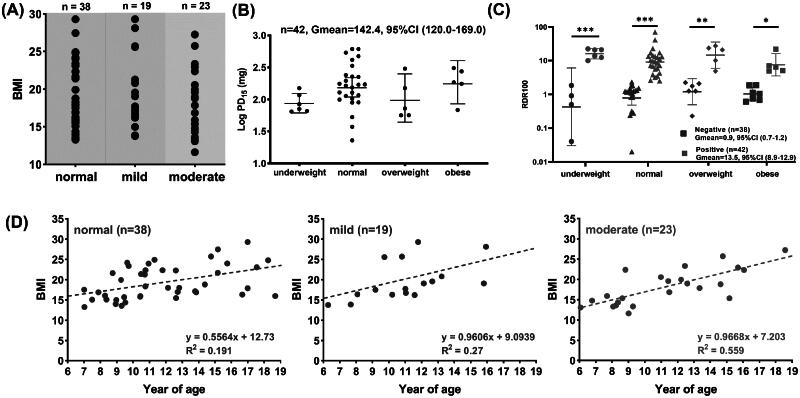
(A) BMI Values of patients with different level of AHR. (B) The average PD_15_ and (C) RDR100 for different BMI class, and (D) the variation of BMI with age for patients with different AHR severity. Significant differences are indicated by asterisks (*p ≤ .05, **p ≤ .01 and ***p ≤ .001).

The average BMI of patients in the normal, mild and moderate AHR were 19.3 ± 4.2, 19.4 ± 4.6 and 18.1 ± 4.4, respectively, with no statistical difference detected (*p* > .05) as depicted in Figure 3A. Figure 3B shows the log PD_15_ dose of patients with positivity AHR in different BMI categories (underweight, normal, overweight and obese). No statistical difference in the PD_15_ dose was noted among all groups. A similar observation was noted for the RDR100 of patients (Figure 3C). However, it was noted that the difference between the positive and negative responses to the mannitol ­challenge test become narrower in the overweight and obese subjects. According to Figure 3D, the BMI values increased as patients become older with a deeper slope noted in patients having moderate AHR (slope = 0.9668, *R*^2^ = 0.559) and mild AHR (slope = 0.9606, *R*^2^ = 0.270), compared with those with no AHR (slope = 0.5564, *R*^2^ = 0.191).

## Discussion

AHR is a significant feature of asthma, and bronchoprovocation testing is frequently adopted to support a diagnosis of asthma and assess treatment outcomes. The present study assessed the association between BMI and asthma control, as reflected by the indirect AHR by mannitol challenge test in a paediatric asthmatic population.

The current degree of AHR of recruited paediatric asthma patients was evaluated with the mannitol challenge test. Half of the recruited subjects tested positive, suggesting their asthma was not fully controlled (Figure 1). This number was distinctly higher than the percentage of patients self-rated with uncontrolled asthma based on the ACT/cACT scores (4%). Previous studies also showed that patients with normal lung function and controlled asthma (based on ACT) could stilled associated with a high degree of AHR in both adult and paediatic populations [37,38]. However, the large discrepancy between the two tests could also be attributed to the difference between the self-perception of symptoms from patients and their actual clinical status.

Higher rates of positive response to the mannitol challenge test were noted in subjects who were receiving ICS medication and concomitantly suffering from allergic rhinitis (Table 1). These results were not completely surprising. It is because patients requiring ICS treatment for asthma control usually have more severe airway inflammation and more frequent/severe asthmatic symptoms [39] and allergic rhinitis is a significant risk factor for developing asthma and an important cause of non-optimal control of asthma [40]. Previous studies demonstrated that asthmatic patients after weeks of daily ICS treatment could abolish their airway hyperresponsiveness to mannitol, possibly due to the attenuation of airway inflammation [34,41]. The higher positive response rates to the mannitol challenge test noted in the present study may hint the poor treatment compliance in patients prescribed with ICS and a close monitoring of their ICS usage may be needed.

Patients with mild AHR showed comparable lung functions as those with no AHR (Figure 2). This agrees with previous findings that active and symptomatic asthma patients could still have normal lung functions [42]. In contrast, Mogensen et al. [35] evaluated the association between uncontrolled asthma and airflow obstruction and reported that uncontrolled asthma is associated with lower FEV_1_/FVC during childhood and early adulthood. It is worth noting that subjects had moderate AHR associated with a significantly lower FEV_1_ and FEV_1_/FVC than those who had no or mild AHR (Figure 2A). It is possibly because the more severe AHR detected by indirect stimuli indicates an elevated airway inflammation, more likely to be reflected in poorer lung functions.

In evaluating the relationship between BMI and AHR, the methacholine challenge test has been the mainstay bronchial provocation test. While multiple studies reported a positive association between BMI and the severity of AHR [26,27], BMI was negatively associated with AHR and asthma symptoms in others [25,28]. Still, obesity was a risk factor for asthma and wheezing. Kwon et al. [28] attributed the inconsistent results to two main aspects: (i) the heterogeneity of the study populations, such as the different ratio of asthmatic to non-asthmatic patients, sex distribution, and proportion of overweight/obese subjects, and (ii) whether asthma was diagnosed based on questionnaires or bronchial provocation tests. While these factors are valid, a key factor overlooked is that the methacholine challenge test is a form of non-specific bronchoprovocation. In other words, numerous medical conditions apart from asthma, such as allergy, sinusitis, smoking, bronchitis and other respiratory infections, could also lead to positive methacholine challenge results [43]. In the non-asthmatic population, AHR was also found to increase with increasing BMI, which is believed to be mediated by small airway closure [21,44]. Other studies also reported the reduction of FEV_1_/FVC with increasing BMI [28,36], regardless of asthmatic and non-asthmatic subjects. Similar findings were noted in the present study (Figure 2B).

Based on the different associations noted between the asthmatic (negative association) and non-asthmatic (positive association) subjects with increasing BMI, Burgess et al. [21] suggested the mechanisms underlying AHR caused by elevated BMI and asthma detected by methacholine challenge test may be different and not additive. To minimise the non-specific contribution of elevated BMI on the AHR of asthmatic patients, mannitol was used as the provocation agent to evaluate patient’s asthma control in the present study. Inhaled mannitol causes bronchial responsiveness due to increased airway osmolarity, an indirect stimulus mediated by effects on inflammatory cells [33]. Therefore, it is generally considered a better index of airway inflammation and variable AHR. The collected data is expected to understand the influence of BMI on the asthma-associated AHR severity. According to our results, BMI had no significant impact on the AHR positivity of patients (Table 1) and no correlation was noted between BMI and AHR severity in paediatric asthmatic patients (Figure 3). This finding was differed from a recent report showing that PD_15_ were positively associated with BMI z-score in a prospective cohort study with twenty-three asthmatic patients aged 4–16 years [45]. The difference could be attributed to the even smaller sample size in that study (only eight and five patients were tested positive with the mannitol challenge test before and after 3-month ICS treatments, respectively). An absence of a relationship between BMI and AHR due to mannitol obtained in the present study suggests the association of obesity with asthma noted in the epidemiological studies are not caused by the enhanced airway inflammation. This is in accord with previous findings that chronic systemic inflammation caused by obesity is different from asthma; it is not eosinophilic or Th2-type inflammation [46]. Collectively, the findings from the present study supported the theory that the more severe asthma in the obese populations is largely attributed to mechanical factors, such as increased chest and abdominal fat mass, instead of elevated airway inflammation.

Research has shown BMI increased with age [47]. Although no strong correlation was found between BMI and AHR, patients with mild and moderate AHR had a steeper increase in BMI with age (Figure 3D), suggesting that overweight/obese asthmatics have a trend to have poorer asthma control at older age than lean ones. However, only a small number of patients ≥15 patients were recruited in the present study that this observation would require further validation in a large-scale study.

### Study limitation

There are several limitations in this study. First, the number of children in the examination groups was not similar. In addition, the recruited subjects were selected based on a history of asthma who had stable asthma (i.e. not in the stage of exacerbation when the functional parameters would have been reduced) at the time of recruitment without special selection of patients who were overweight or obese. The majority of the recruited subjects were in the normal weight range (58.8%) that the powering may be bias to this group, leading to a high chance of a Type II error. Second, the mannitol test kit required patients to actuate the device with designated inhalation effort. Although the training was provided to patients prior to the testing, the different capabilities of patients aerosolising the mannitol powder may lead to inaccurate PD_15_ dose determination, particularly for younger patients who had more difficulty in aerosolising powders at the highest dose (2–4 breaths were generally required to empty a 40 mg mannitol capsule). Despite this, the positive responses to the mannitol challenge test aligned with the clinical features of asthma (currently receiving ICS treatments and concomitant allergic rhinitis). Thirdly, BMI measurement was the only approach of ascertaining the metabolic condition of subjects in this study. Recent data showed that reduced skeletal muscle mass, often noted in obese individuals, may be associated with an increased risk of exacerbation in asthma patients [48,49]. The relationship between skeletal muscle mass and AHR may provide additional information on the impacts of metabolic condition on asthma control. Last but not least, the present cross-sectional study only measured the AHR levels of patients at a single time point, which makes it difficult to reflect the level of asthma control over a period of time, given that the airway conditions of asthmatic patients may vary due to different factors, like season, exposure to trigger agents, and medication use, etc. The impacts of these factors were minimised by excluding patients with recent (within four weeks) exacerbation and respiratory infections from our study.

## Conclusion

“Indirect” AHR determined by the mannitol challenge test were evaluated in pediatric asthmatic patients. A high prevalence of AHR (>50%) was found in those self-evaluated with good asthma control using the ACT. No significant correlation between BMI and AHR was found in the present study, suggesting that the more severe asthma noted in the obese populations is more likely due to mechanical factors instead of airway inflammatory. This implies that increasing ICS treatment for obese asthmatic patients with poor response to asthmatic medication may not be a practical and effective approach. However, it is worth noting that our observation was based on a small sample of overweight/obese subjects, further verification is required in a larger population.

## Data Availability

All data that supports the findings in this study, including clinical data, are available from the corresponding author upon reasonable request.
